# Genetic Silencing of Pacemaker Cells: Local Intervention With Global Implications

**DOI:** 10.1161/JAHA.112.001412

**Published:** 2012-04-24

**Authors:** Caline S. Karam, Fadi G. Akar

**Affiliations:** Cardiovascular Institute, Mount Sinai School of MedicineNew York, NY

**Keywords:** biological pacemaker, gene therapy, heart failure, heart rate, Ivabradine

## Introduction

Heart failure is a major public health epidemic that claims hundreds of thousands of lives annually in the United States alone. Despite important advances in the management and prevention of many cardiovascular disorders, the incidence and prevalence of heart failure are increasing with the aging of the population. Existing treatments for patients with heart failure are far from being optimal and are often associated with proarrhythmic activity and risk of sudden cardiac death. Therefore, the search for novel, effective, and safe therapeutic options for these patients represents a critical area of unmet need for this country and the rest of the world. In an elegant proof of concept study published in this issue of the *Journal of the American Heart Association (JAHA)*, Lugenbiel et al^1^ describe a novel gene-based approach for potentially treating heart failure by targeting discrete cell populations within the sino-atrial node.

Several lines of evidence derived from experimental and clinical studies indicate that a controlled heart rate is an important target for heart failure therapy.^2^ In fact, a strong link between average heart rate and cardiovascular morbidity in patients with heart failure has been documented. In clinical trials the ability of a given heart failure treatment to reduce overall mortality is, in some cases, predicted by its heart rate lowering efficacy. Moreover, a causative relation between elevated heart rate and heart failure is epitomized by the fact that sustained tachycardia, in the absence of other risk factors or insults, is a well-established mechanism of ventricular remodeling that culminates in the onset of heart failure.^3^ In the experimental laboratory, numerous groups have exploited the relation between elevated heart rate and left ventricular dysfunction by developing highly reproducible small and large animal models of heart failure using chronic tachycardia pacing of the right atrium or ventricle. These experimental models have been instrumental in advancing our fundamental understanding of the mechanisms that underlie mechanical, structural, electrical, metabolic, ionic, and molecular dysfunction in the failing heart. Of note, cessation of rapid pacing following the onset of heart failure is typically associated with reversal of pathological electrical and structural remodeling and an improved cardiac function,^4^ further highlighting the role of heart rate control in the management of heart failure.

Hallmark features of the failing heart include a prominent reduction in contractile efficiency and the loss of contractile reserve. These deficits, caused by altered calcium signaling, excitation-contraction coupling, and decreased myofilament sensitivity to calcium can lead to hemodynamic insufficiency, compromise, and death. To enhance cellular contractility, β-adrenergic receptor agonists, such as isoproterenol and dobutamine, have been developed. The positive inotropic property of these agents is mediated, in part, by the enhancement in activator calcium through L-type calcium channels which are phosphorylated by cAMP-dependent protein kinase A. Unfortunately the chronic use of these agents has been severely hampered by adverse effects, including heart rate acceleration and promotion of ventricular arrhythmias.^5,6^ Indeed, chronic β-adrenergic receptor stimulation, which increases the energetic cost of contractility at a time of reduced oxygen supply, can exacerbate myocardial damage and accelerate mortality in heart failure patients. In contrast, β-adrenergic receptor blockers, which are mainstays of heart failure therapy, are distinguished by their heart rate lowering efficacy and their ability to hinder and/or reverse key features of pathological remodeling in the failing heart. Because β-adrenergic receptor blockers are confounded by negative inotropic properties that can further reduce the contractile function of an already compromised failing heart, their efficacy in the acute and subacute phases is often limited. To that end, considerable interest in recent years has been directed toward the development of specific heart rate lowering agents that selectively target pacemaker channels. Ideally, such agents would mimic the beneficial effects of β-blockers in terms of heart rate reduction while avoiding their confounding side effects on contractility.

Ivabradine, a selective inhibitor of the pacemaker current (*I_f_*), was developed to suppress heart rate by targeting the hyperpolarization-activated cyclic nucleotide (HCN) gene family. In experimental and clinical studies, ivabradine improved myocardial energetics and left ventricular function. Specifically, in the SHIFT trial (Systolic Heart failure treatment with *I_f_* Inhibitor ivabradine), patients with symptomatic heart failure who were treated with ivabradine had a significant benefit (by >25%) in terms of cardiovascular mortality and hospitalization when compared with the placebo treated group.^7^ These highly encouraging findings have cemented the concept of heart rate modulation through pacemaker channel inhibition as an important strategy for the management of heart failure.^2,8,9^ As with any pharmacological therapy for a chronic disease, challenges related to dosing, bioavailability, selectivity, and adverse interactions with other drugs are expected. These complicating issues warrant further optimization, as alternative nonpharmacological approaches for modulating heart rate are explored.

In this issue of *JAHA*, Lugenbiel et al^1^ present an exciting alternative to the use of ivabradine for lowering heart rate. These authors systematically develop and test a novel, gene-based approach that selectively targets pacemaker channels by silencing the stimulatory G protein coupled receptor alpha subunit (G_αs_), specifically in the sino-atrial node. In effect, these authors use highly sophisticated molecular and gene therapy tools to create a “biological beta blocker” that can be locally delivered.

In what follows, we provide a brief overview of recent efforts to develop cell- and gene-based therapies for heart rate control, including the creation of “bio-pacemakers” for combating bradycardia^10^ and “bio-beta blockers” for combating tachycardia.^11^ This is followed by a discussion of the unique advantages and potential pitfalls of the exciting approach undertaken by Lugenbiel et al^1^ for suppressing heart rate.

## Gene Therapies for Bradycardia: Biological Pacemakers

Major milestones have been recently achieved in our collective effort to develop novel, gene-based approaches for the treatment of cardiac rhythm disorders. Indeed, several groups have made great strides toward the creation of biological pacemakers that can one day (perhaps in the near future) either fully replace or at the very least assist existing electronic devices.^10,12–14^ Our growing understanding of the molecular basis of cardiac pacemaking activity, including the revelation of a complex interplay between voltage and calcium clocks within the cardiac myocyte, has been instrumental in the identification of key targets that can be exploited for the creation of viable biological pacemakers.

In proof of concept studies, Miake et al^14^ used viral gene transfer of an engineered dominant negative construct of Kir2.1 (Kir2.1AAA) to focally inhibit the inward rectifier potassium current; thereby converting normally quiescent cells into ones that exhibited spontaneous depolarization and rhythmic activity. When injected into the left ventricle, these constructs could “capture” the myocardium resulting in premature ventricular beats that likely emanated from the site of injection.^14^ In other seminal studies, Rosen et al systematically developed HCN based approaches for the creation of robust biological pacemakers, both in vitro and in vivo.^10,12,13,15–17^ These biological pacemakers provided physiologically acceptable heart rates and exhibited positive chrontropic responses to emotional arousal and stress. Indeed, the efficacy and translatability of these HCN-based approaches were elegantly documented in preclinical large animal models by the Rosen group.^10,12,13,15–17^

## Gene Therapies for Tachycardia: Biological β-Blockers

As mentioned earlier, heart rate reduction is an important target for heart failure therapy. As such, novel molecular tools that can either lower resting heart rate or dampen the excessive acceleration of heart rate upon β-adrenergic stimulation are needed. To that end, Donahue and colleagues pioneered efforts to create gene therapy tools for rhythm disorders by targeting the L-type calcium current (*I_Ca-L_*),^11,18^ which mediates action potential propagation in specialized conducting tissues, such as the sino-atrial and atrio-ventricular nodes.

*I_Ca-L_*, whose activity is strongly modulated by cAMP-dependent PKA activity is activated by G_αs_ and inhibited by G_αi_. As such, partial *I_Ca-L_* blockade can theoretically be achieved either by the overexpression of G_αi_ or the silencing of G_αs_. In a landmark study, Donahue et al used G_αi_ overexpression to suppress baseline atrioventricular conduction and slow heart rate during atrial fibrillation,^11^ providing compelling evidence that myocardial gene transfer can be effectively used to manage the most common tachyarrhythmia in man.

In this issue of *JAHA*, an exciting complementary approach for heart rate reduction was developed and tested in structurally normal pigs.^1^ Specifically, the authors tested whether selective silencing of G_αs_ in the region of the sinoatrial node using a targeted adenoviral-mediated gene transfer approach coupled with an electroporation technique can effectively lower heart rate. They provided compelling data that reducing the expression levels of G_αs_ using siRNA causes a significant reduction in the heart rate of animals 7 days postgene transfer. In addition, they showed that this approach does not elicit an adverse effect on myocardial contractility. These exciting findings raise a number of issues. One obvious question is whether this approach can effectively control heart rate in the setting of heart failure, as these studies were conducted in normal myocardium. This issue might be of particular importance considering the marked remodeling of HCN transcripts that occurs in the setting of heart failure. Indeed, Nattel and colleagues^19,20^ documented important changes in the expression levels of various HCN isoforms in the sino-atrial node and in atrial and ventricular rmyocardium in a canine model of pacing-induced heart failure. These molecular changes likely promote sinus node dysfunction and increased frequency of atrial and ventricular ectopy. In light of these findings, one must question whether *selective* inhibition of pacemaker activity in the sinus node is a superior strategy over global *I_f_* inhibition, as the former might tip the balance in favor of atrial and ventricular ectopy. If so, does the targeted therapy proposed by Lugenbiel et al^1^ achieve rate control at the expense of a disrupted rhythm? Does altering the sequence of myocardial activation by potentially unleashing ventricular ectopic foci exacerbate electrical remodeling and predispose to malignant arrhythmias?

Furthermore, a clinically relevant strategy will warrant chronic (not acute) suppression of heart rate. This surely cannot be achieved by a short-term adenoviral mediated gene transfer strategy coupled with a highly invasive electroporation technique. The development of clinically translatable gene delivery methods and cardiotropic vectors for long-term modulation of gene expression in the sino-atrial node and/or the myocardium would be required. Indeed, the use of various adeno-associated vector serotypes or adeno-associated vector chimera may prove essential in achieving this ambitious aim. As with any good study, the intriguing findings by Lugenbiel et al^1^ leave us with more questions than answers. In the coming years, these important questions will surely be addressed by these investigators and others.
